# Supplementary material for: Optogenetic relaxation of actomyosin contractility uncovers mechanistic roles of cortical tension during cytokinesis
Source: Nat Commun. 2021 Dec 8;12:7145. doi: 10.1038/s41467-021-27458-3 (PMC8654997; doi:10.1038/s41467-021-27458-3)
Supplement: Supplementary file 1 — Supplementary Information [file 41467_2021_27458_MOESM1_ESM.pdf]

Supplementary Information

Optogenetic relaxation of actomyosin contractility uncovers mechanistic roles of cortical tension during cytokinesis.

Author list

Kei Yamamoto[1,2,3], Haruko Miura[1,2], Motohiko Ishida[4,5], Yusuke Mii[1,2,3,6], Noriyuki Kinoshita[1,3], Shinji Takada[1,2,3], Naoto Ueno[1,3,7], Satoshi Sawai[4,5], Yohei Kondo[1,2,3,8*], and Kazuhiro Aoki[1,2,3,7,8*]

Affiliations

[1]National Institute for Basic Biology, National Institutes of Natural Sciences, 5-1 Higashiyama, Myodaiji-cho, Okazaki, Aichi 444-8787, Japan.

[2]Exploratory Research Center on Life and Living Systems (ExCELLS), National Institutes of Natural Sciences, 5-1 Higashiyama, Myodaiji-cho, Okazaki, Aichi 444-8787, Japan.

[3]Department of Basic Biology, School of Life Science, SOKENDAI (The Graduate University for Advanced Studies), 5-1 Higashiyama, Myodaiji-cho, Okazaki, Aichi 444-8787, Japan.

[4]Graduate School of Arts and Sciences, University of Tokyo, Komaba, 153-8902 Tokyo, Japan.

[5]Research Center for Complex Systems Biology, Universal Biology Institute, University of Tokyo, Komaba, 153-8902 Tokyo, Japan.

[6]Japan Science and Technology Agency (JST), PRESTO, 4-1-8 Honcho, Kawaguchi, Saitama, 332-0012, Japan.

[7]IRCC International Research Collaboration Center, National Institutes of Natural Sciences, 4-3-13 Toranomon, Minato-ku, Tokyo 105-0001, Japan.

[8]These authors jointly supervised this work.

*Correspondence: y-kondo@nibb.ac.jp or k-aoki@nibb.ac.jp

Supplementary Note 1

Physical modeling

Previous studies have established a simple equation for the equilibrium of forces between the contractile ring and the two polar cortices (Yoneda and Dan 1972; Sedzinski et al. 2011; Turlier et al. 2014). Based on the Young-Laplace law, the force balance is written as

$$\sigma_{\mathrm{r}} = 2R_{\mathrm{r}}T_{\mathrm{c}} \cos\theta \text{ , (S1)}$$

where σ_{r} is the net tension in the ring and T_{c} is the tension in the polar cortices (see Supplementary Figure 8 for the definitions of the geometric factors R_{r} and θ). Since the turnover of cortical actin (~10 sec) is much faster than the time scale of cytokinesis (~10 min), the material property of polar cortices can be described as active viscous liquid (Turlier et al. 2014). Thus, the cortical tension depends on both the mechanical strength of the cortical cytoskeleton, which causes the effective viscosity, and myosin motors, which cause active tension. Here we simply describe the cortices as an active viscous membrane having tension T_{c}. On the other hand, the net tension in the ring is composed of two factors, tension generated by myosin motors T_{r} and viscous resistance by, e.g., cross-linkers, as

$$\sigma_{\mathrm{r}} = T_{\mathrm{r}} + \alpha \frac{dR_{\mathrm{r}}}{dt}, \text{ (S2)}$$

where the coefficient α represents the viscosity of the actin network constituting the ring (Sedzinski et al. 2011). Here, if we combine Eqs. S1 and S2, we obtain

$$\alpha \frac{dR_{\mathrm{r}}}{dt} = -(T_{\mathrm{r}} - 2R_{\mathrm{r}}T_{\mathrm{c}} \cos\theta) \text{ , (S3)}$$

This is Eq. (1) in the main text.

Lower bound of the cortical tension

In our model, the furrow ingression rate of control cells (Control-light and OptoMYPT-dark), v, can be expressed as

$$v \propto F_{\mathrm{r}} - F_{\mathrm{c}} \text{ , (S4)}$$

where F_{r} and F_{c} are the ring tension and the cortical tension, respectively (Fig. S8). This is Eq. (2) in the main text. The furrow ingression rate of OptoMYPT-pole cells, v', can be expressed as

$$v' \propto F_{\mathrm{r}} - F_{\mathrm{c}}' \text{ , (S5)}$$

where F_{c}' is the cortical tension upon blue light illumination. Note that F_{r} is considered to be constant under each condition, because blue light was locally illuminated to the polar cortices. Taken together with Eqs. S4 and S5, we obtain

$$v/v' = (F_\mathrm{r} - F_\mathrm{c})/(F_\mathrm{r} - F_\mathrm{c}') > (F_\mathrm{r} - F_\mathrm{c})/(F_\mathrm{r}). \quad \text{(S6)}$$

The rightmost expression represents the case where blue light illumination completely reduces cortical tension to zero. We rearrange the above formula as

$$F_\mathrm{c}/F_\mathrm{r} > 1 - v/v'. \quad \text{(S7)}$$

Based on our experimental data in Figure 4, the furrow ingression rate of Control-pole cells, OptoMYPT-dark cells, and OptoMYPT-pole cells were v = 1.55 ± 0.20 µm/min, v = 1.93 ± 0.33 µm/min, and v'= 2.26 ± 0.25 µm/min, respectively. Thus, the cortical tension relative to ring tension is estimated as

$$F_\mathrm{c}/F_\mathrm{r} > 1 - (1.55\sim1.93)/2.26 = 0.15\sim0.31. \quad \text{(S8).}$$

Supplementary Figures

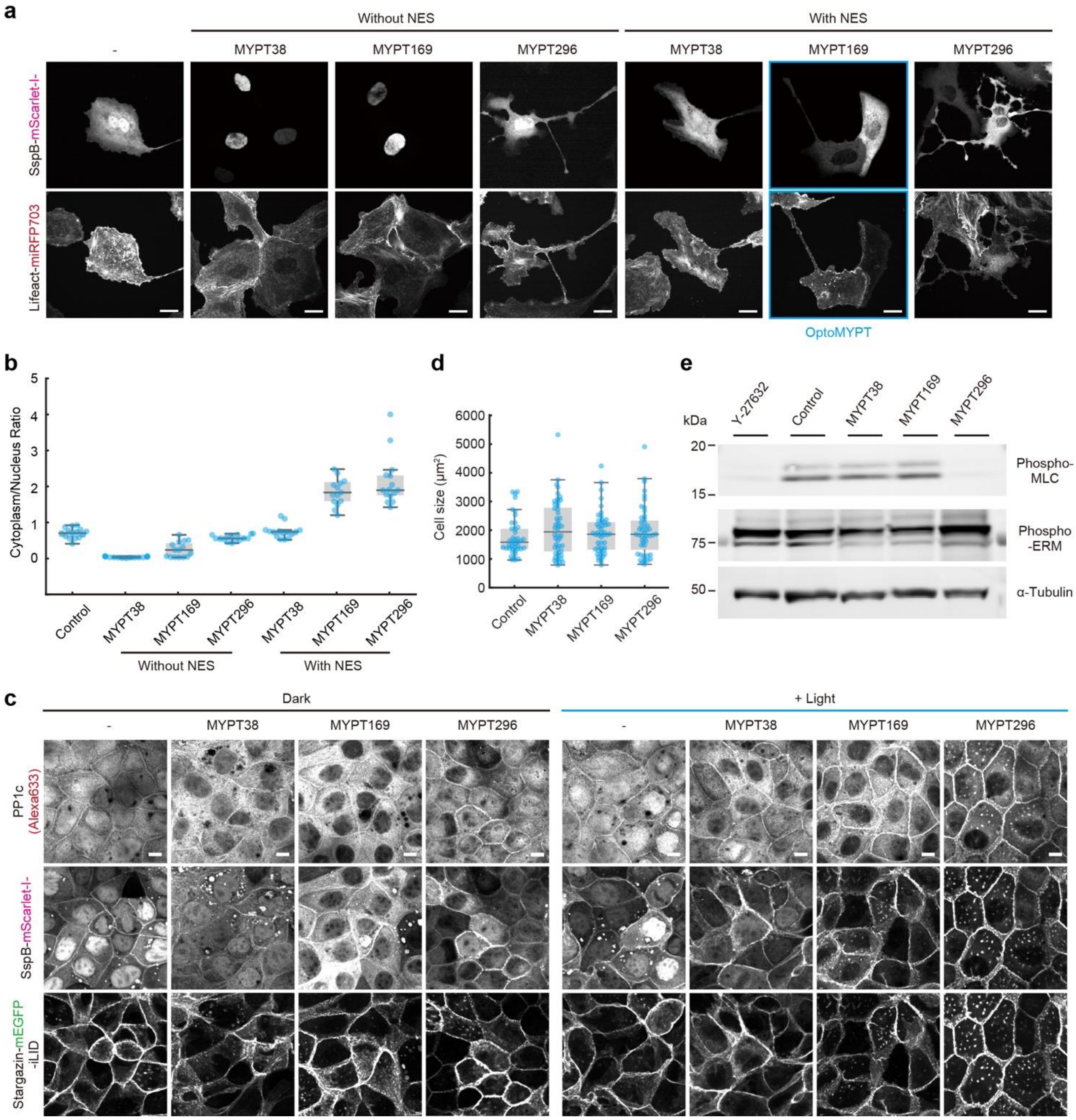

Supplementary Figure 1. Optimization of the length of PP1BD for OptoMYPT.
(a) Representative images of MDCK cells transiently expressing SspB-mScarlet-I or SspB-mScarlet-I-PP1BDs without or with NES (upper panels), transiently expressing Stargazin-mEGFP-iLID, and stably expressing Lifeact-miRFP703 (lower panels). SspB-mScarlet-I-MYPT169-NES was used for the OptoMYPT (panels outlined in blue). Of note, SspB-mScarlet-I-MYPT296-expressing cells showed aberrant morphology with elongated protrusions. Scale bar, 20 µm. (b) The ratio of cytoplasmic to nuclear fluorescence intensity of SspB-mScarlet-I-MYPTs was quantified in each cell, and shown as a box plot. A blue dot indicates data from individual cells. n = 15 cells for each condition. (c) Immunofluorescence of the endogenous PP1c in MDCK cells stably expressing SspB-mScarlet-I-PP1BDs with NES, and Stargazin-mEGFP-iLID. In the four columns at right, blue light was illuminated from the top of the culture dishes for 3 min before fixation. Scale bar, 20 µm. (d) The cell size of MDCK cells transiently expressing SspB-mScarlet-I or SspB-mScarlet-I-

PP1BDs with NES was quantified, and shown as a box plot as in panel b. n = 41, 48, 48, and 47 for cells expressing plasmids of Control, MYPT38, MYPT169, and MYPT296, respectively. (e) Western blot analysis of phospho-MLC, phospho-ERM, and α-Tubulin in MDCK cells stably expressing SspB-mScarlet-I (Control) or SspB-mScarlet-I-PP1BDs with NES, and Stargazin-mEGFP-iLID (representative from three independent experiments). Control cells were treated with 40 μM Y-27632 as a negative control (Y-27632).

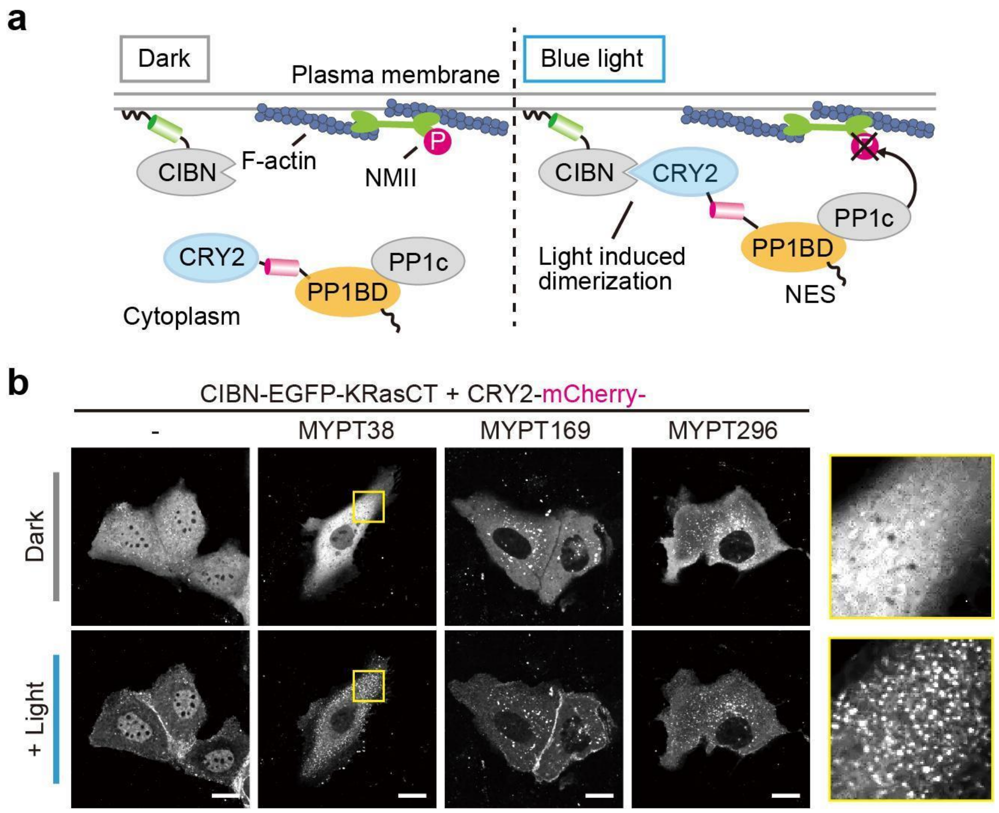

Supplementary Figure 2. CRY2-based OptoMYPT system.
(a) Schematics of the CRY2-based OptoMYPT system. (b) Representative images of MDCK cells transiently expressing CRY2-mCherry or CRY2-mCherry-PP1BDs with NES, and CIBN-EGFP-KRasCT (representative from three independent experiments). The cells expressing CRY2-mCherry-MYPT38 showed aggregates and puncta upon blue light illumination (yellow boxed region). Blue light was illuminated through the objective lens for 500 msec every 1 min. Scale bar, 20 μm.

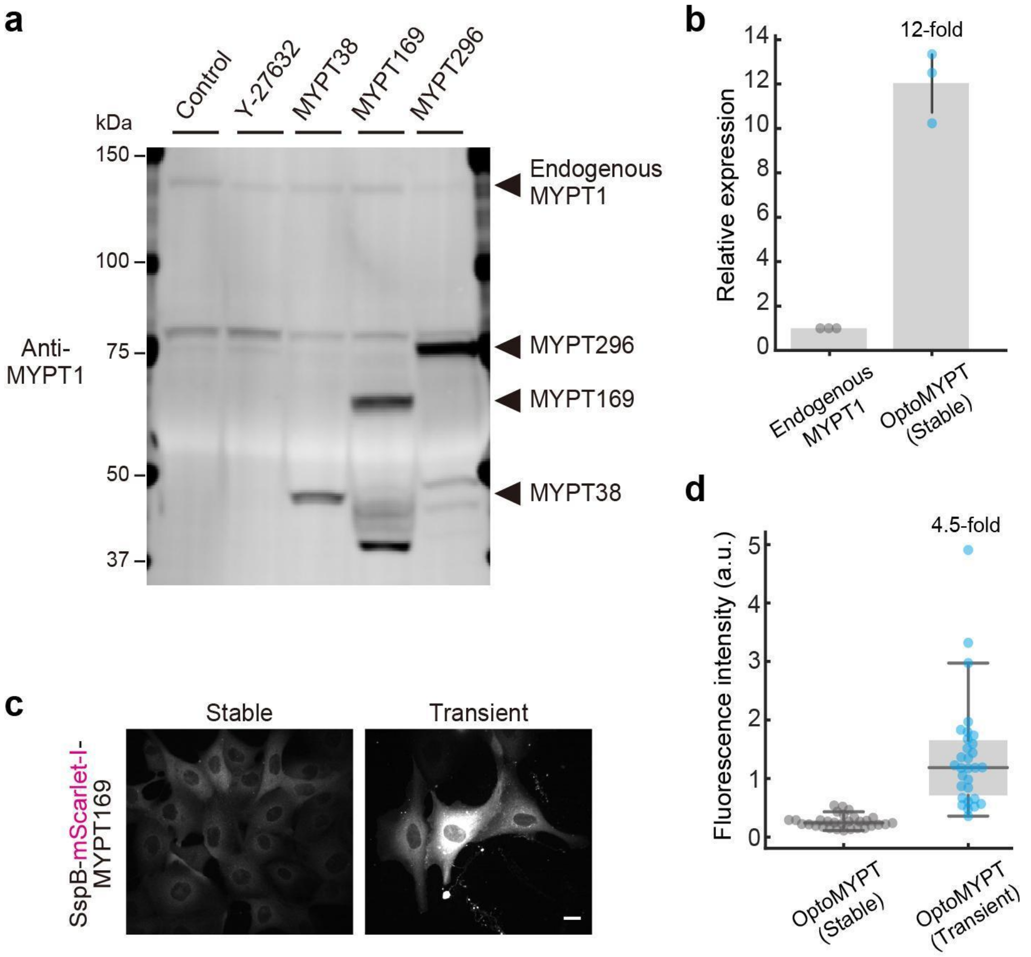

Supplementary Figure 3. Quantification of the expression level of OptoMYPT relative to endogenous MYPT1.

(a) Western blot analysis of MYPT1 in MDCK cells stably expressing SspB-mScarlet-I (Control) or SspB-mScarlet-I-PP1BDs with NES, and Stargazin-mEGFP-iLID. Control cells were treated with 40 µM Y-27632 as a negative control (Y-27632). Note that the MYPT1 antibody recognizes the N-terminus of the MYPT1. (b) Quantification of the relative expression level of SspB-mScarlet-I-MYPT169 to endogenous MYPT1. A total of n = 3 experiments were performed and data are presented as mean +/− SD. (c, d) The representative images of SspB-mScarlet-I-MYPT169 in stably and transiently expressing MDCK cells are shown (c). The expression levels of SspB-mScarlet-I-MYPT169 in stably and transiently expressing MDCK cells are represented as a box plot, in which the box extends from the first to the third quartile with the whiskers denoting 1.5 times the interquartile range. A blue dot indicates data from individual cells. n = 30 cells for each condition. Scale bar, 20 µm.

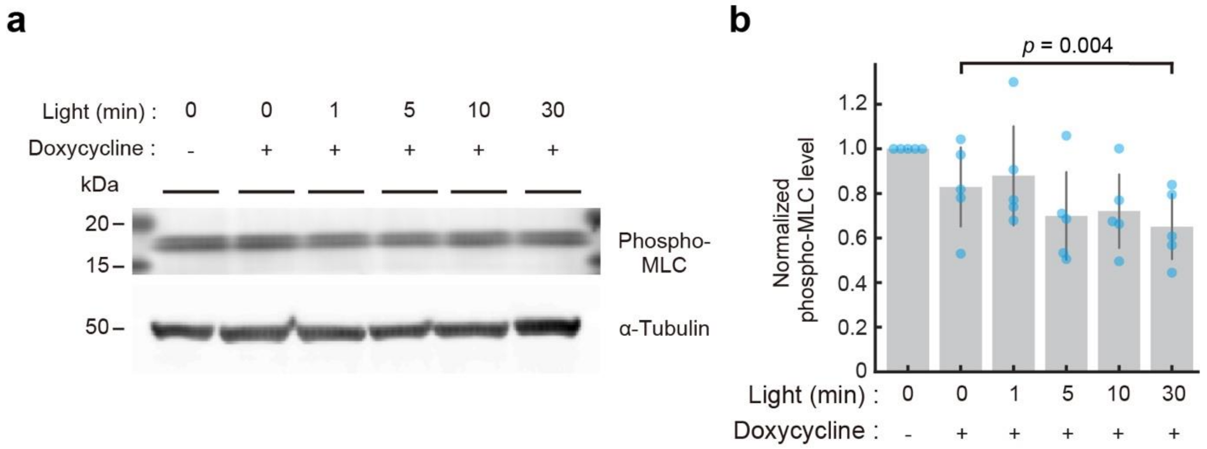

Supplementary Figure 4. OptoMYPT-induced dephosphorylation of MLC with Western blot analysis.

(a) Western blot analysis of phosphorylated MLC and α-Tubulin in MDCK cells. In this cell line, Stargazin-mEGFP-iLID is constitutively expressed, whereas SspB-mScarlet-I-MYPT169 is expressed in a doxycycline-dependent manner (0.25 µg/mL doxycycline for one day). Blue light was globally and continuously illuminated from the top of the dish for the number of minutes indicated on the figure. (b) Quantification of phosphorylated MLC levels. α-Tubulin was used as loading control. A total of n = 5 experiments were performed and data are presented as mean +/– SD. $p = 0.004$ (Paired *t*-test).

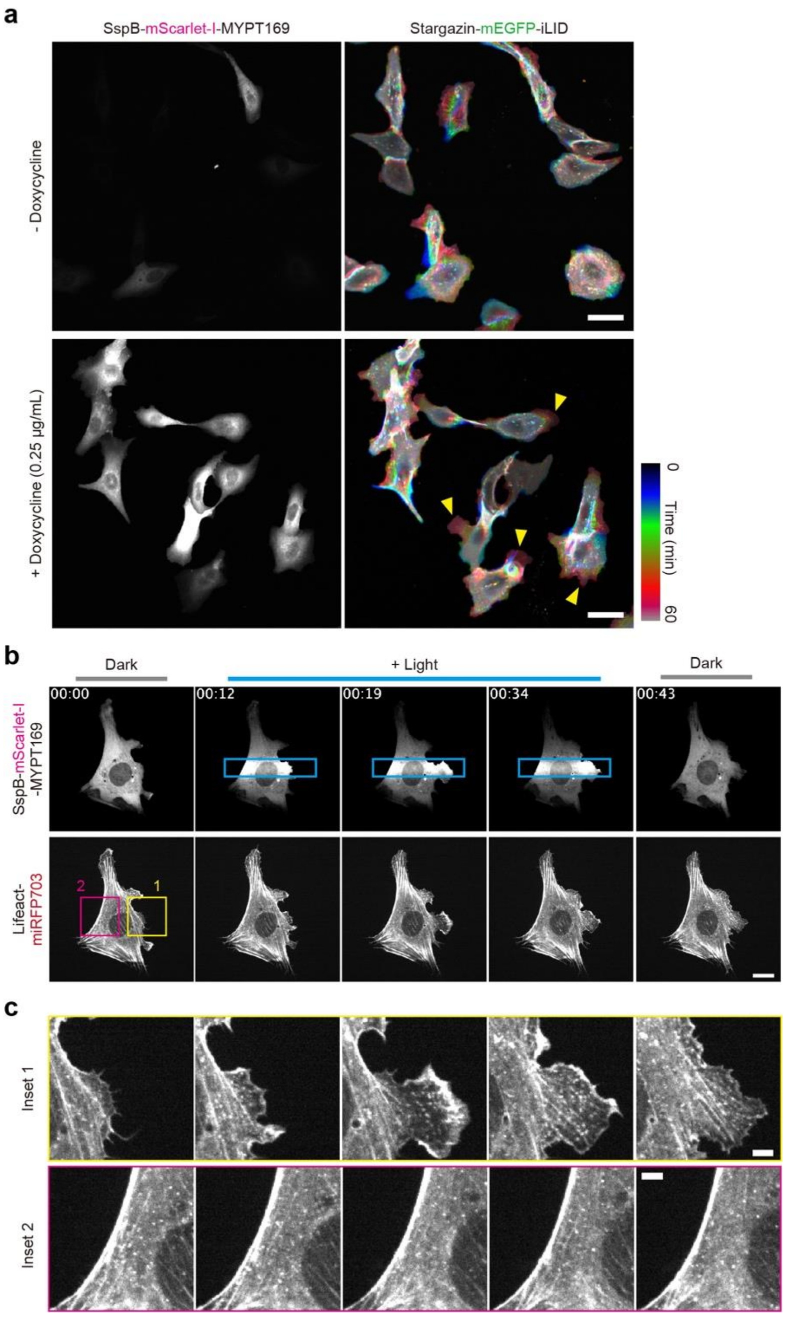

Supplementary Figure 5. OptoMYPT-induced membrane protrusions.
(a) Simultaneous visualization of SspB-mScarlet-I-MYPT169 with Stargazin-mEGFP-iLID in MDCK cells (representative from two independent experiments). In this cell line, Stargazin-mEGFP-iLID is constitutively expressed, whereas SspB-mScarlet-I-MYPT169 is transiently expressed by a doxycycline-inducible promoter (0.25 µg/mL doxycycline for one day). Blue light was globally and continuously illuminated from the top of the dish just after imaging onset for 60 min. The upper and lower panels represent the cells without or with doxycycline induction, respectively. The left columns show the expression of SspB-mScarlet-I-MYPT169, indicating the induction of SspB-mScarlet-I-MYPT169 in a doxycycline-dependent manner. The right columns show the deformation of cell shape across 60 min of blue light illumination, with the time elapsed indicated by the color code on the right side. Yellow arrowheads indicate lamellipodial protrusion. Scale bar, 50 µm. (b) Simultaneous visualization of SspB-mScarlet-I-MYPT169 (upper) with F-actin (Lifeact-miRFP703) (lower) in NIH-3T3 cells (representative of 4 cells from two independent experiments). The rectangular regions were illuminated with blue light for 500 msec every 20 sec. Blue rectangles indicate illuminated areas. Scale bar, 20 µm. (c) Inset images of Lifeact-miRFP703 in panel b. Scale bar, 5 µm.

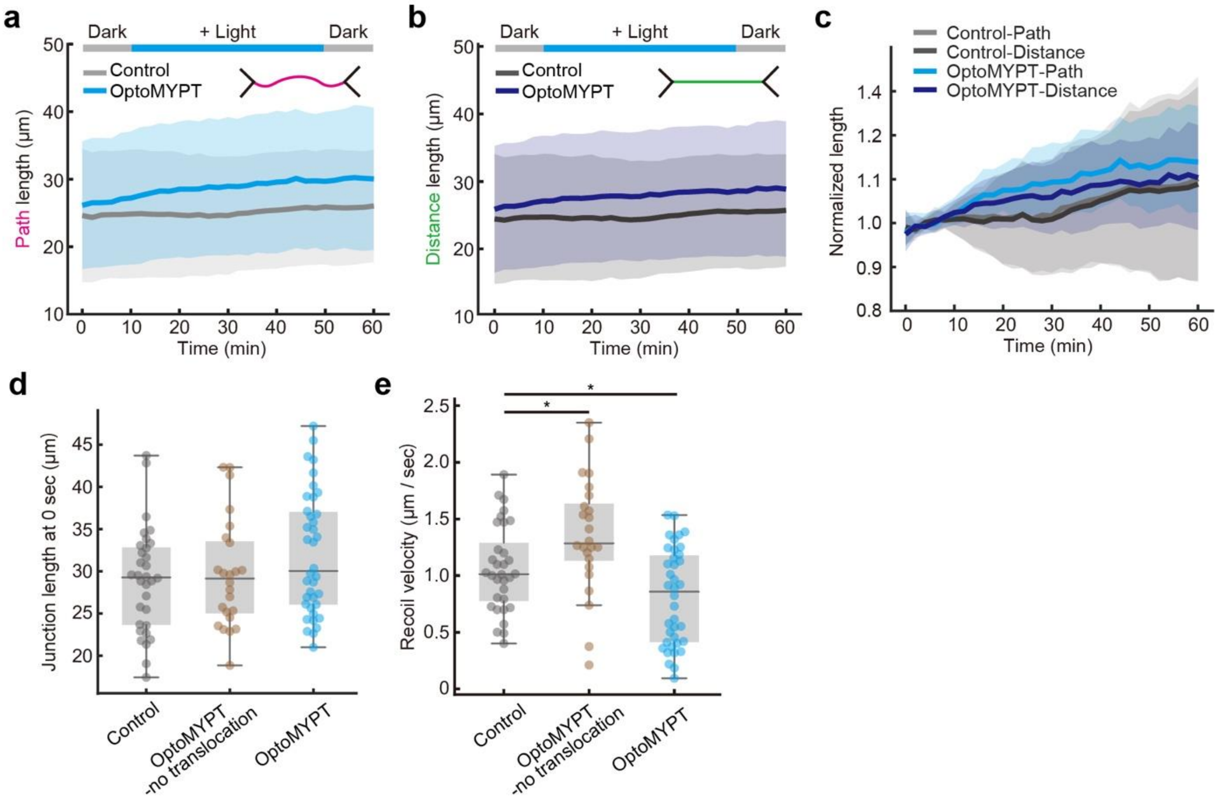

Supplementary Figure 6. OptoMYPT reduced the cell-cell junctional tension in *Xenopus* embryos.

(a, b) The path length (a) and total distance (b) of cell-cell junctions were quantified in *Xenopus* embryos expressing SspB-mScarlet-I (Control) and SspB-mScarlet-I-MYPT169 (OptoMYPT) with Stargazin-mEGFP-iLID. The average of path length and total distance are plotted as a function of time with SD. Results are shown for n = 15 cells from three embryos for both Control and OptoMYPT. (c) The path length and total distance in panel a and b were normalized by the average value before blue light illumination, and are plotted as a function of time with SD. (d, e) The junction length at t = 0 sec (d) and the recoil velocity (e) are shown as a box plot. A dot indicates data from an individual cell. Recoil velocity was calculated from the difference in junction length before (t = 0) and just after (t = 3.08) laser ablation. Results are shown for n = 32, 24, and 38 cells from 6 embryos examined over 2 independent batches for Control, OptoMYPT-no translocation, and OptoMYPT, respectively. *p = 0.018 and 0.013 for Control-OptoMYPT-no-translocation and Control-OptoMYPT, respectively (two-tailed student's t-test).

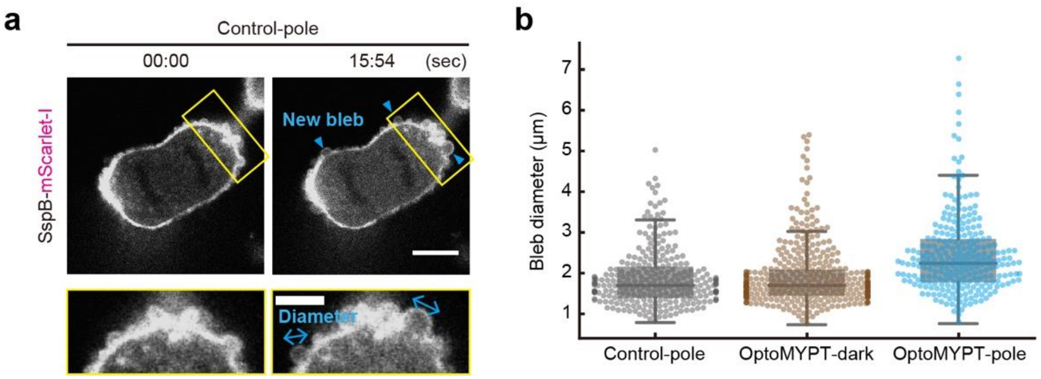

Supplementary Figure 7. Quantification of the bleb diameter.
(a) Representative image of an MDCK cell expressing SspB-mScarlet-I and Stargazin-mEGFP-iLID during cytokinesis. The blue arrowheads in the upper panel indicate new blebs per 15.54 sec. The blue arrowheads in the lower panel indicate measured bleb diameters. The lower panels are a magnified view of the yellow-boxed regions in the upper panels. Scale bar, 10 and 5 μm for the upper and lower panels, respectively. (b) The diameter of blebs in the Control-pole, OptoMYPT-dark, and OptoMYPT-pole cells are shown as a box plot with a swarm plot. The counted number of blebs was 266, 360, and 257 for Control, OptoMYPT-dark, and OptoMYPT-pole, respectively.

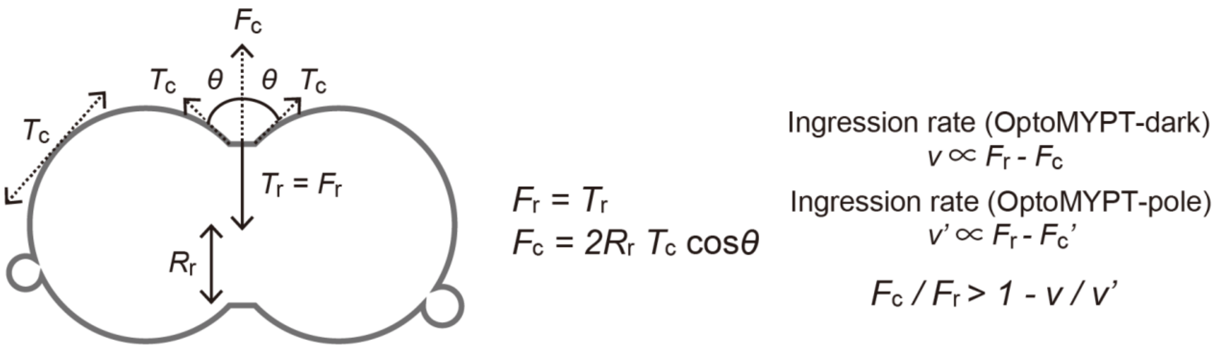

Supplementary Figure 8. Physical modeling.
T_c and T_r are the cortical tensions at the actin cortex and contractile ring, respectively. R_r is the ring diameter. F_r and F_c correspond to the force generated by ring tension and cortical tension, respectively.

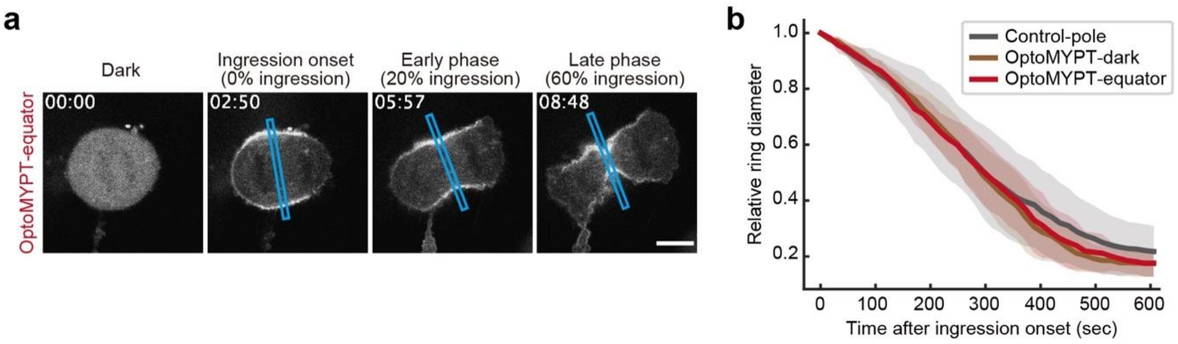

Supplementary Figure 9. Blue light illumination to the equatorial region during cytokinesis.
(a) Representative images of SspB-mScarlet-I-MYPT169 in MDCK cells during cytokinesis. The blue boxed regions were illuminated with blue light every 3.11 sec. Scale bar, 10 µm. (b) Quantification of the furrow ingression rate in OptoMYPT-equator cells. Ingression rates of Control-pole and OptoMYPT-dark in Figure 4f are merged. Averaged relative diameters are plotted as a function of time with the SD. Results are shown for n = 5 cells for OptoMYPT-equator.

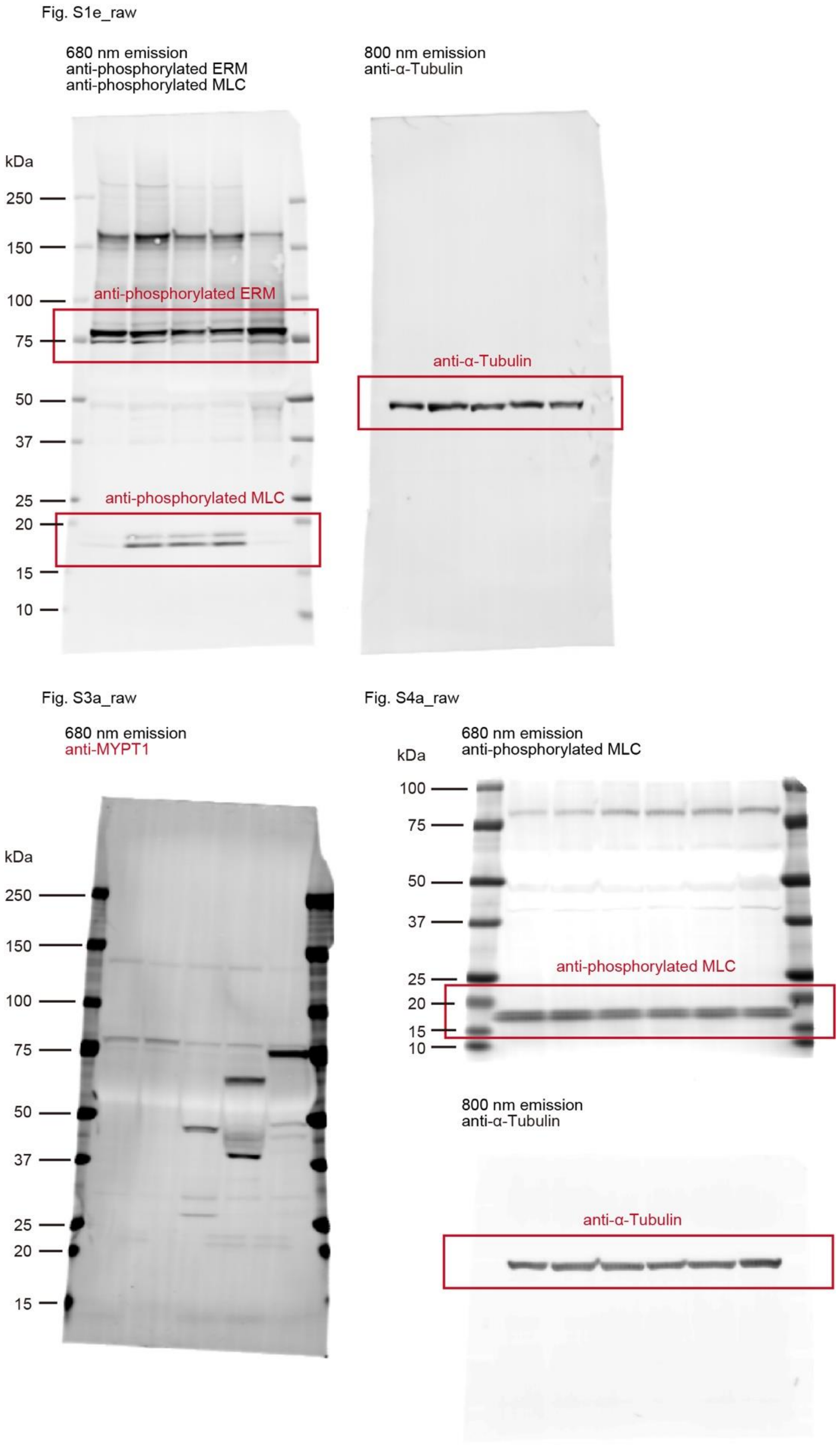

Supplementary Figure 10. Original images for western blots.
The original images for the results of Supplementary Figure 1e, 3a, and 4a.

Supplementary Table

Plasmid name	Relevant features	Figures	Source or reference	Sequence
pCAGGS-SspB-mScarlet-I	CAG promoter, SspB-mScarlet-I	1d, 2d,f, 3b, 4b,c, S1a,c, S7a	This study	https://benchling.com/s/seq-LizO8aJvYQvytA26tnvk
pCAGGS-SspB-mScarlet-I-MYPT38	CAG promoter, SspB-mScarlet-I-MYPT38-NES	1d, S1a,c	This study	https://benchling.com/s/seq-e5c4XNGJU7VUDY1Adw0D
pCAGGS-SspB-mScarlet-I-MYPT169	CAG promoter, SspB-mScarlet-I-MYPT169-NES	1d, 2e,f,h, 3c, 4b,c, 5a, S1a,c, S3c, S5b,c, S9a	This study	https://benchling.com/s/seq-VFtIByTEhOa69PaBJ7FF
pCAGGS-SspB-mScarlet-I-MYPT296	CAG promoter, SspB-mScarlet-I-MYPT296-NES	1d, S1a,c	This study	https://benchling.com/s/seq-NfHUqB2v72pMzw7Umbrv
pCAGGS-SspB-mScarlet-I-MYPT38 w/o NES	CAG promoter, SspB-mScarlet-I-MYPT38 (without NES)	S1a	This study	https://benchling.com/s/seq-M5vohEavn7YouFTOsbqR
pCAGGS-SspB-mScarlet-I-MYPT169 w/o NES	CAG promoter, SspB-mScarlet-I-MYPT169 (without NES)	S1a	This study	https://benchling.com/s/seq-4cIRYdnnkc5w1D7EG5pN
pCAGGS-SspB-mScarlet-I-MYPT296 w/o NES	CAG promoter, SspB-mScarlet-I-MYPT296 (without NES)	S1a	This study	https://benchling.com/s/seq-ik7oQqR9I1hNqtdzAjGK
pCAGGS-Stargazin-mEGFP-iLID	CAG promoter, Stargazin-mEGFP-iLID	1d, 2d-f,h, 3b,c, 4b,c, 5a, S1a,c, S3c, S5b,c, S7a, S9a	This study	https://benchling.com/s/seq-XUhPNN7CavWnbGAq1TUe
pCAGGS-PP1c-miRFP703	CAG promoter, PP1c-miRFP703	1d	This study	https://benchling.com/s/seq-8370yYN7igj1pyuf3TeZ
pCAGGS-CRY2-mCherry	CAG promoter, CRY2-mCherry	2b, S2b	This study	https://benchling.com/s/seq-bhM5JFtyaGL3itlXyRYw
pCAGGS-CRY2-mCherry-MYPT38	CAG promoter, CRY2-mCherry-MYPT38-NES	S2b	This study	https://benchling.com/s/seq-tLDYRgH228MpcEprZ8H3
pCAGGS-CRY2-mCherry-MYPT169	CAG promoter, CRY2-mCherry-MYPT169-NES	2b, S2b	This study	https://benchling.com/s/seq-jlDVZxO6I2j5bRqXLNAe
pCAGGS-CRY2-mCherry-MYPT296	CAG promoter, CRY2-mCherry-MYPT296-NES	S2b	This study	https://benchling.com/s/seq-KgPOub2LM388hRapgVNp
pCAGGS-CIBN-EGFP-KRasCT	CAG promoter, CIB N-terminus-EGFP-KRasCT	2b, S2b	This study	https://benchling.com/s/seq-RA16GvytRbNXBNzKX1hg
pPBbsr2-Lifeact-miRFP703	PiggyBac transposase donor vector, Lifeact-miRFP703	2d-f,h, S1a, S5b,c	This study	https://benchling.com/s/seq-6Umf325oqmk6HOmbykIX
pPBpuro-SspB-mCherry	PiggyBac transposase donor vector, SspB-mCherry	S1c,e, S3a	This study	https://benchling.com/s/seq-5d7Oj31YcZlxJqSsnNAa
pPBpuro-SspB-mCherry-MYPT38	PiggyBac transposase donor vector, SspB-	S1c,e, S3a	This study	https://benchling.com/s/seq-fZysCS2LhI69PbhGD4tg

	mCherry-MYPT38-NES			
pPBpuro-SspB-mCherry-MYPT169	PiggyBac transposase donor vector, SspB-mCherry-MYPT169-NES	S1c,e, S3a,c	This study	https://benchling.com/s/seq-RrmRNQx2r9ULkAGO3yxd
pPBpuro-SspB-mCherry-MYPT296	PiggyBac transposase donor vector, SspB-mCherry-MYPT296-NES	S1c,e, S3a	This study	https://benchling.com/s/seq-WFNiQng3AysDppDUz4fk
pPBbsr2-Stargazin-mEGFP-iLID	PiggyBac transposase donor vector, Stagazin-mEGFP-iLID	S1c,e, S3a,c	This study	https://benchling.com/s/seq-iKCLTeMJ2f84JqYhfn1n
pPBbsr2-rtTA2-TRE-SspB-mScarlet-I-MYPT169	PiggyBac transposase donor vector, Doxycycline inducible expression, SspB-mScarlet-I-MYPT169	S4a, S5a	This study	https://benchling.com/s/seq-xf8zdl8VLNsyQHRmCXqC
pT2Apuro-Stargazin-mEGFP-iLID	Stargazin-mEGFP-iLID	S4a, S5a	This study	https://benchling.com/s/seq-rBh93iqQQT5hBXFNIIVG
pCAGGS-T2TP	CAG promoter, Tol2 transposase	S4a, S5a	This study	https://benchling.com/s/seq-yGo10mX966NnMTfpmt3F
pCAGGS-hyPBase	CAG promoter, PiggyBac transposase	2d-f,h, S1c,e, S3a,c, S4a, S5a-c	This study	https://benchling.com/s/seq-oGkw53b41IZqvzF5yQ9K
pCSf107mT-Lifeact-miRFP703	mRNA synthesis vector for *Xenopus*, Lifeact-miRFP703	3e,f	This study	https://benchling.com/s/seq-vtY7pCfmSVMpUQZXF5Us
pCSf107mT-SspB-mScarlet-I	mRNA synthesis vector for *Xenopus*, SspB-mScarlet-I	3e,h	This study	https://benchling.com/s/seq-NMiukyuyg4J64MgeMSrG
pCSf107mT-SspB-mScarlet-I-MYPT169	mRNA synthesis vector for *Xenopus*, SspB-mScarlet-I-MYPT169	3e,h	This study	https://benchling.com/s/seq-j1NmidSuKAuAX6LzGTy6
pCSf107mT-Stargazin-mEGFP-iLID	mRNA synthesis vector for *Xenopus*, Stargazin-mEGFP-iLID	3e,h	This study	https://benchling.com/s/seq-town7d8HHoJTrkapTNKX
pCSf107mT-mEGFP-KRasCT	mRNA synthesis vector for *Xenopus*, mEGFP-KRasCT	3h	This study	https://benchling.com/s/seq-0kClMWXvr2A6LllNkxtP

Supplementary Table 1. Plasmids used in this study.
