# Supplementary material for: Optogenetic relaxation of actomyosin contractility uncovers mechanistic roles of cortical tension during cytokinesis
Source: Nat Commun. 2021 Dec 8;12:7145. doi: 10.1038/s41467-021-27458-3 (PMC8654997; doi:10.1038/s41467-021-27458-3)
Supplement: Supplementary file 12 — Reporting Summary [file 41467_2021_27458_MOESM12_ESM.pdf]

nature portfolio

Corresponding author(s): Kazuhiro AOKI

Last updated by author(s): Nov 17, 2021

Reporting Summary

Nature Portfolio wishes to improve the reproducibility of the work that we publish. This form provides structure for consistency and transparency in reporting. For further information on Nature Portfolio policies, see our Editorial Policies and the Editorial Policy Checklist.

Statistics

For all statistical analyses, confirm that the following items are present in the figure legend, table legend, main text, or Methods section.

n/a	Confirmed	
☐	☒	The exact sample size (*n*) for each experimental group/condition, given as a discrete number and unit of measurement
☐	☒	A statement on whether measurements were taken from distinct samples or whether the same sample was measured repeatedly
☐	☒	The statistical test(s) used AND whether they are one- or two-sided<br>*Only common tests should be described solely by name; describe more complex techniques in the Methods section.*
☒	☐	A description of all covariates tested
☒	☐	A description of any assumptions or corrections, such as tests of normality and adjustment for multiple comparisons
☐	☒	A full description of the statistical parameters including central tendency (e.g. means) or other basic estimates (e.g. regression coefficient) AND variation (e.g. standard deviation) or associated estimates of uncertainty (e.g. confidence intervals)
☐	☒	For null hypothesis testing, the test statistic (e.g. *F*, *t*, *r*) with confidence intervals, effect sizes, degrees of freedom and *P* value noted<br>*Give P values as exact values whenever suitable.*
☒	☐	For Bayesian analysis, information on the choice of priors and Markov chain Monte Carlo settings
☒	☐	For hierarchical and complex designs, identification of the appropriate level for tests and full reporting of outcomes
☒	☐	Estimates of effect sizes (e.g. Cohen's *d*, Pearson's *r*), indicating how they were calculated

Our web collection on statistics for biologists contains articles on many of the points above.

Software and code

Policy information about availability of computer code

Data collection	MetaMorph (ver 7.10.3), LAS X (ver 3.5.5)
Data analysis	Fiji/ImageJ (ver 2.1.0/1.53c), Python (ver 3.7), Microsoft Excel (ver 16.54)

For manuscripts utilizing custom algorithms or software that are central to the research but not yet described in published literature, software must be made available to editors and reviewers. We strongly encourage code deposition in a community repository (e.g. GitHub). See the Nature Portfolio guidelines for submitting code & software for further information.

Data

Policy information about availability of data

All manuscripts must include a data availability statement. This statement should provide the following information, where applicable:

- Accession codes, unique identifiers, or web links for publicly available datasets
- A description of any restrictions on data availability
- For clinical datasets or third party data, please ensure that the statement adheres to our policy

Source data are provided with this paper. Full western blot images are in Supplementary Figure 10. Imaging data generated in this study have been deposited in the RIKEN SSBD:repository (Systems Science of Biological Dynamics repository) with the doi: https://doi.org/10.24631/ssbd.repos.2021.11.002. The plasmids would be available from Addgene. The nucleotide sequences of newly generated constructs including oligonucleotides for PCR and DNA sequences are provided in Supplementary Table 1. Source data for figures and supplementary information are provided with the paper. T

Field-specific reporting

Please select the one below that is the best fit for your research. If you are not sure, read the appropriate sections before making your selection.

- [x] Life sciences
- [ ] Behavioural & social sciences
- [ ] Ecological, evolutionary & environmental sciences

For a reference copy of the document with all sections, see nature.com/documents/nr-reporting-summary-flat.pdf

Life sciences study design

All studies must disclose on these points even when the disclosure is negative.

Sample size	Sample size is shown in the figure, figure legends, or in the methods section. No statistical method like power analysis was used to pre-determined sample size. Sample sizes were limited by practically and throughput, and are consistent with previously published similar works in the field.
Data exclusions	No data were excluded.
Replication	All experiments were performed with independent replicates as described in the figure legends.
Randomization	No randomization was performed, because randomization was not relevant to our study using cultured cells and Xenopus embryos.
Blinding	No blind test was performed, as is standard practice in the field.

Reporting for specific materials, systems and methods

We require information from authors about some types of materials, experimental systems and methods used in many studies. Here, indicate whether each material, system or method listed is relevant to your study. If you are not sure if a list item applies to your research, read the appropriate section before selecting a response.

Materials & experimental systems

n/a	Involved in the study	
[ ]	[x]	Antibodies
[ ]	[x]	Eukaryotic cell lines
[x]	[ ]	Palaeontology and archaeology
[ ]	[x]	Animals and other organisms
[x]	[ ]	Human research participants
[x]	[ ]	Clinical data
[x]	[ ]	Dual use research of concern

Methods

n/a	Involved in the study	
[x]	[ ]	ChIP-seq
[x]	[ ]	Flow cytometry
[x]	[ ]	MRI-based neuroimaging

Antibodies

Antibodies used	ppMLC antibody (1:500 dilution; Cell Signaling Technology #3674)<br>phospho-Ezrin/Radixin/Moesin antibody (1:500 dilution, Cell Signaling Technology #3276)<br>MYPT1 antibody (1:500 dilution; Cell Signaling Technology #2634)<br>PPP1CB antibody (1:200 dilution; abcam #ab53315)<br>α-Tubulin antibody (DM1A) (1:5000 dilution; sc-32293: Santa Cruz Biotechnology)<br>IRDye680LT-conjugated goat polyclonal anti-rabbit IgG (H + L) (1:5000 dilution; LI-COR Bioscience #925-68021)<br>IRDye800CW-conjugated donkey polyclonal anti-mouse IgG (H + L) (1:5000 dilution; LI-COR Bioscience #925-32212)<br>Alexa 633-conjugated anti-rabbit IgG (1:1000 dilution; ThermoFisher)
Validation	All antibodies are validated by vendor using Western blots. In addition to the vendor validations for the antibodies listed above, the study investigators validated each antibody with positive control reference samples with known reactivities. The antibodies all generally performed as expected.

Eukaryotic cell lines

Policy information about cell lines

Cell line source(s)	MDCK cells were from RIKEN (no. RCB0995: RIKEN Bioresource Center) . NIH-3T3 cells were from RIKEN (no. RCB0150: RIKEN Bioresource Center). HeLa cells, which were a kind gift from Michiyuki Matsuda (Kyoto University), were from ATCC (CCL-2).
Authentication	The identity of the MDCK, NIH-3T3, and HeLa cell lines was not authenticated independently by the study investigators.

Mycoplasma contamination	The study investigators did not assay the MDCK and NIH-3T3 cell lines used in this study for mycoplasma. HeLa cells were tested negative for mycoplasma contamination.
Commonly misidentified lines (See ICLAC register)	No commonly misidentified cell line was used in this study.

Animals and other organisms

Policy information about studies involving animals; ARRIVE guidelines recommended for reporting animal research

Laboratory animals	Xenopus laevis frogs from our colony provided eggs (females) and testis (males). Females roughly range from 2 to 8 years old and males roughly range from 1 to 3 years old.
Wild animals	No wild animals were used in the study.
Field-collected samples	No samples were collected from the field.
Ethics oversight	Embryos used in this study were obtained from a colony of Xenopus laevis frogs maintained at the National Institute for Basic Biology under the care of the Division of Molecular and Developmental Biology. All experiments using Xenopus laevis were approved by The Institutional Animal Care and Use Committee, National Institutes of Natural Sciences (Permit Number 18A038, 19A062, 20A053).

Note that full information on the approval of the study protocol must also be provided in the manuscript.
