# Supplementary material for: Optogenetic relaxation of actomyosin contractility uncovers mechanistic roles of cortical tension during cytokinesis
Source: Nat Commun. 2021 Dec 8;12:7145. doi: 10.1038/s41467-021-27458-3 (PMC8654997; doi:10.1038/s41467-021-27458-3)
Supplement: Supplementary file 13 — Description of Additional Supplementary Files [file 41467_2021_27458_MOESM13_ESM.pdf]

Title: Supplementary Movie 1.
Description: Membrane translocation of SspB-mScarlet-I-MYPT169 and PP1c-miRFP upon global blue light illumination. MDCK cells expressing SspBmScarlet-I-MYPT169 (left) or PP1cmiRFP703 (right) with Stargazin-mEGFPiLID were imaged with global blue light stimulation. Scale bar, 20 µm.

Title: Supplementary Movie 2.
Description: Induction of membrane protrusion by the OptoMYPT system. An MDCK cell expressing SspBmScarlet-I (upper) or SspB-mScarlet-IMYPT169 (lower) together with StargazinmEGFP-iLID and Lifeact-miRFP703 was stimulated with blue light as indicated by the cyan rectangle. Scale bar, 20 µm.

Title: Supplementary Movie 3.
Description: Induction of membrane retraction on the opposite side of the blue-light illuminated area in an MDCK cell. An MDCK cell expressing SspBmScarlet-I-MYPT169 (left) together with Stargazin-mEGFP-iLID and LifeactmiRFP703 (right) were stimulated with blue light as indicated by the cyan rectangle. Scale bar, 20 µm.

Title: Supplementary Movie 4.
Description: Induction of membrane protrusion by global blue light illumination in MDCK cells expressing the OptoMYPT system. MDCK cells were treated without (left) or with (right) doxycycline to induce SspB-mScarlet-IMYPT169. Stargazin-mEGFP-iLID was imaged for 60 min with global blue light illumination from the top of the culture dishes. Scale bar, 20 µm.

Title: Supplementary Movie 5.
Description: Induction of membrane protrusion by the OptoMYPT system in an NIH-3T3 cell. Blue rectangles indicate blue-light illuminated areas. Scale bar, 20 µm.

Title: Supplementary Movie 6.
Description: Traction force measurement upon blue light illumination. An MDCK cell expressing SspB-mScarlet-I (left) or SspB-mScarlet-I-MYPT169 (right) with Stargazin-mEGFP-iLID was seeded on polyacrylamide gel containing infra-red fluorescent beads. The images of mScarlet-I (upper) and pseudo color traction force (lower) are represented. Blue rectangles indicate blue-light illuminated areas. Scale bar, 20 µm.

Title: Supplementary Movie 7.
Description: Membrane translocation of SspB-mScarlet-I proteins in Xenopus embryos. Xenopus embryos expressing SspB-mScarlet-I (upper left) or SspB-mScarlet-I-MYPT169 (lower left) together with Stargazin-mEGFP-iLID and Lifeact-miRFP703 (right column) were imaged with global blue light stimulation. Scale bar, 20 µm.

Title: Supplementary Movie 8.
Description: Laser ablation experiments at the cell-cell junction in Xenopus embryos. The cell-cell junctions in the animal pole region of Xenopus embryos were ablated by laser at t = 0 sec under Control (left), OptoMYPT-no translocation (middle), and OptoMYPT (right) conditions. Scale bar, 20 µm.

Title: Supplementary Movie 9.
Description: Cytokinesis of a pole-illuminated MDCK cell expressing the OptoMYPT system. MDCK cells expressing SspB-mScarlet-I (Control-pole, left) or SspB-mScarlet-I-MYPT169 (OptoMYPTdark, middle; OptoMYPT-pole, right) and Stargazin-mEGFP-iLID were imaged with or without blue light illumination at the both poles. Of note, the Control-pole cell shows slight mitotic oscillation, especially in the later phase. Scale bar, 10 µm.

Title: Supplementary Movie 10.

Description: Cytokinetic shape oscillation of a single poleilluminated MDCK cell expressing SspBmScarlet-I-MYPT169 and StargazinmEGFP-iLID. The cyan circle and magenta arrow indicate the blue light illumination point and large bleb, respectively. Scale bar, 10 µm.
